# Effect of Bis(maltolato)oxovanadium(IV) on Zinc, Copper, and Manganese Homeostasis and DMT1 mRNA Expression in Streptozotocin-Induced Hyperglycemic Rats

**DOI:** 10.3390/biology11060814

**Published:** 2022-05-25

**Authors:** Cristina Sánchez-González, Laura Moreno, Pilar Aranda, María Montes-Bayón, Juan Llopis, Lorenzo Rivas-García

**Affiliations:** 1 Department of Physiology, Institute of Nutrition and Food Technology ’’José Mataix“, Biomedical Research Centre, University of Granada, Avda. Del Conocimiento s.n., 18100 Armilla, Spain; crissg@ugr.es (C.S.-G.); laura.moreno.terron@gmail.com (L.M.); paranda@ugr.es (P.A.); jllopis@ugr.es (J.L.); 2 Sport and Health Research Centre, University of Granada, C/. Menéndez Pelayo 32., 18016 Armilla, Spain; 3 Department of Physical and Analytical Chemistry, Faculty of Chemistry, University of Oviedo, 33007 Oviedo, Spain; montesmaria@uniovi.es

**Keywords:** mineral metabolism, DMT1, nutrition

## Abstract

**Simple Summary:**

Diabetes mellitus is a disease that affects some metabolic processes, altering the metabolism of trace elements associated with the antioxidant defense system. The insulin-mimetic activity of vanadium has received attention in recent years. However, the use of vanadium as an antidiabetic agent is limited due to its pro-oxidant properties, which are probably caused by interactions with other trace elements. There is currently little information on the interactions between vanadium and zinc, copper and manganese in diabetic rats. For these reasons, the objective of this study was to examine whether diabetic rats treated with bis(maltolato)oxovanadium(IV) (BMOV(IV)) undergo alterations in zinc, copper and manganese homeostasis, and whether such changes are related to the gene expression of the divalent metal transporter 1 (DMT1). The results obtained from this study showed that the metabolism of Zn, Cu and, to a lesser extent, Mn is altered by diabetes and that BMOV(IV) administered to diabetic rats at a dose of 3 mg V/day reverted the alterations in zinc and copper homeostasis produced by diabetes. The effect of this supplementation was possibly mediated by a decrease in food intake and DMT1 gene expression.

**Abstract:**

Our aim was to examine whether vanadium (IV) corrects alterations in zinc, copper and manganese homeostasis, observed in streptozotocin-induced hyperglycemic rats, and whether such changes are related to divalent metal transporter 1 (DMT1) mRNA expression, and antioxidant and proinflammatory parameters. Four groups of Wistar rats were examined: control; hyperglycemic (H); hyperglycemic treated with 1 mg V/day (HV); and hyperglycemic treated with 3 mg V/day (HVH). Vanadium was supplied in drinking water as bis(maltolato)oxovanadium(IV) for five weeks. Zinc, copper and manganese were measured in food, excreta, serum and tissues. DMT1 mRNA expression was quantified in the liver. Hyperglycemic rats showed increased Zn and Cu absorption and content in the liver, serum, kidneys and femurs; DMT1 expression also increased (*p* < 0.05 in all cases). HV rats showed no changes compared to H rats other than decreased DMT1 expression (*p* < 0.05). In the HVH group, decreased absorption and tissular content of studied elements (*p* < 0.05 in all cases) and DMT1 expression compared to H (*p* < 0.05) were observed. Liver zinc, copper and manganese content correlated positively with glutathione peroxidase activity and negatively with catalase activity (*p* < 0.05 in both cases). In conclusion, treatment with 3 mg V/d reverted the alterations in zinc and copper homeostasis caused by hyperglycemia, possibly facilitated by decreased DMT1 expression.

## 1. Introduction

Hyperglycemia is a common consequence of uncontrolled diabetes, which, over time, leads to severe damage to several body systems, altering the concentration of trace elements and enzymes associated with the antioxidant defense system [1,2]. Consequently, this may contribute to the exacerbation of the disease.

Zinc (Zn), copper (Cu) and manganese (Mn) are three widely distributed minerals with an important role in the correct functioning of the organism and are related to the divalent metal transporter 1 (DMT1) [3]. Zinc, essentiality, is associated with its role as a cofactor for more than 300 enzymes, playing an important role in several biochemical pathways [4,5]. Moreover, Zn is involved in the cell division process, DNA and RNA synthesis, the expression of specific genes and the systemic regulation of the immune system. Furthermore, Zn has been associated with insulin synthesis and action. Thus, the decrease in pancreatic Zn levels was associated with an increase in reactive oxygen species (ROS) [6,7]. Zn also acts on the insulin signaling pathway in skeletal muscle cells, stimulating glucose oxidation [4]. In addition, other roles in diabetes mellitus could be attributed to Zn; thus, Zn improved the glycometabolic control in this condition, mediated by the downregulation of the protein tyrosine phosphatase 1B (PTP1B) [8]. Moreover, Zn inhibited insulin receptor substrates 1 and 2 and consequently reduced oxidative stress [9] and suppressed ROS generation by its action on the superoxide dismutase enzyme (SOD) [10]. Copper is another mineral with diverse implications in glucose metabolism. Thus, a disturbance in Cu levels has been correlated with abnormalities in the metabolic pathways of diabetes mellitus [5]. Other authors described how renal and serum Cu levels in a mice model of type 1 diabetes mellitus were significantly higher than in normal mice, and hepatic Cu and Zn levels were significantly lower [11]. Cu and manganese (Mn) act on several metabolic processes in bone formation, wound healing and antioxidant defense by SOD. In diabetes mellitus, there is marked inflammation and oxidative stress, which has been related to decreased expression and activity of cytoplasmic (Zn, Cu-SOD), mitochondrial (Mn-SOD) and extracellular (Cu-SOD) SOD, an effect observed in different tissues, such as the liver, skeletal muscle and the kidneys in diabetic animal models [12].

Vanadium (V) is an essential element for some living organisms, but its role as a micronutrient has not yet been clarified. Moreover, some aspects of its role in cell metabolism, the toxicological implications and the therapeutic applications remain unknown [13,14,15]. Clarifying the metabolic implication of V in diabetes mellitus has received attention in recent years. Thus, vanadate and vanadyl complexes have demonstrated their capacity to inhibit some complexes, such as P-type ATPases in the insulin signaling cascade [16], to stimulate the cytosolic protein k, and to promote the glucose transporter GLUT4 translocation from the intracellular compartment to the plasmatic membrane [17,18]. Hence, the insulin-mimetic activity of V and the development of a wide variety of organovanadium (IV) complexes has been an advance in the introduction of vanadium pharmaceuticals as insulin-mimetic drugs [17,19]. Thus, bis(maltolato)oxovanadium(IV) (BMOV(IV)) has been reported to be more effective than vanadyl sulfate [VO(SO_4_)] as a glucose-lowering agent and to be better tolerated in animal models [19]. Nevertheless, some aspects of the V interaction with other metallic elements, especially antioxidant metals, remain unclear. The current use of V as an hypoglycemic agent is limited due to its pro-oxidant properties, cytokine production and inflammatory implications [14,20]. Several authors related the pro-oxidant activity of V to other trace elements, such as magnesium (Mg), iron (Fe), copper (Cu), selenium (Se) and/or zinc (Zn) [21,22,23].

There is currently little information about the interactions between V and Zn, Cu and Mn in diabetic rats. In diabetic-streptozotocin (STZ) rats, Oster et al. observed increases in the renal content of Zn and Cu but not Mn, and found that treatment with sodium metavanadate decreased the content of both elements to levels closer to those of control rats but observed no changes in the hepatic levels of Zn and Cu [21]. Similar results have been published for Zn, Cu and Mn in diabetic-STZ rats, where five organic vanadium complexes were tested [24]. However, diabetes did not change the spleen content of these metals, whereas the five complexes tested increased Zn and Cu content in the spleen, but only two complexes increased Mn content in this organ [25].

Nonetheless, other authors have suggested that treatment with BMOV(IV) did not modify the status of other minerals [26]. A decrease in hepatic Zn and Cu content in the liver, kidney and spleen [27] and increased Cu in the femur have been reported in control rats treated with sodium metavanadate [28]. Thus, treating rats with BMOV(IV) did not modify Mn homeostasis [29]. Hence, it is necessary to clarify the role of Zn, Cu and Mn in hyperglycemic rats and their relation with V, in order to develop the possible potential application of this metal in diabetes treatment.

For these reasons, the objective of the present study was to examine whether hyperglycemic rats treated with BMOV(IV) showed altered bioavailability and tissue distribution of Zn, Cu and Mn. It was also investigated whether these changes were related to the expression of DMT1 genes and whether they were associated with the activity of enzymes related to antioxidant defense and proinflammatory parameters.

## 2. Materials and Methods

### 2.1. Animals and Diets

**Figure 1:**
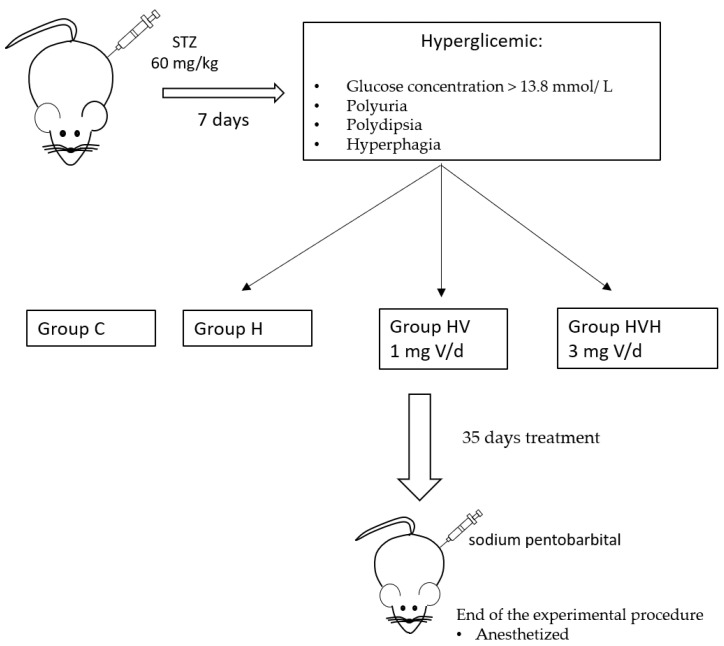
Schematic study design. C (control rats); H (hyperglycemic rats); HV (hyperglycemic rats treated with 1 mg V per day); HVH (hyperglycemic rats treated with 3 mg V per day); STZ (streptozotocin).

Male Wistar rats aged 45–48 days, weighing 190–220 g (Charles River Laboratories, L’Abresde, France) were used. The animals were randomly distributed into four groups. (1) The control group (C) consisted of 9 rats fed with the AIN93M diet (60 µg V, 40.5 mg Zn, 6.3 mg Cu, and 12.8 mg Mn/kg food). (2) The hyperglycemic group (H) consisted of 10 hyperglycemic rats fed with the semisynthetic AIN93M diet. (3). The hyperglycemic group treated with 1 mg V/day (HV) consisted of 10 hyperglycemic rats fed with the semisynthetic AIN93M diet and supplemented with 6.22 mg bis(maltolato)oxovanadium(IV) (BMOV(IV))/day added to drinking water, which supplied 1 mg V/d. (4) The hyperglycemic group treated with 3 mg V/day (HVH) consisted of 10 hyperglycemic rats fed with the semisynthetic AIN93M diet and supplemented with 18.66 mg bis(maltolato)oxovanadium(IV) (BMOV(IV))/day added to drinking water, which supplied 3 mg V/d. Figure 1 shows a schematic representation of the study design. In all cases, hyperglycemia was induced in rats by a single injection of streptozotocin (STZ) at a dose of 60 mg/kg dissolved in a buffer solution with pH 4.5. Preparation was extemporaneous and this was administered by the intraperitoneal route.

After seven days, the criteria for considering the animals as hyperglycemic were as follows: the rats had a glucose concentration > 13.8 mmol/L, and the conditions of polyuria, polydipsia and hyperphagia. Daily, the consumption of water was calculated and the BMOV solution was administered in the drinking water over 35 days. The synthesis method of BMOV was described previously [30]. Finally, on day 35, the rats were anesthetized employing an intraperitoneal injection of sodium pentobarbital (5 mg/100 g body weight) and exsanguinated by the canulation of the posterior aorta. Then, the blood was centrifuged at 1200× *g* during 15 to divide the serum. Subsequently, some tissues such as liver, kidneys, gastrocnemius muscles, spleen, heart and femurs were removed, weighed and placed in pre-weighed polyethylene vials, and stored at −80 °C.

All animals were placed from the beginning of the experiment (day 0) in individual metabolic cages prepared for the separate collection of feces and urine. During the last week of the experimental period (7 days), feces and urine were collected every 24 h and stored at −80 °C in polyethylene bottles for subsequent analysis. The cages were located in a well-ventilated, temperature-controlled room (21 °C), with relative humidity ranging from 40% to 60% and a light:dark period of 12h. The following biological indices were calculated: absorbed as (I − F); absorption (%) as ((I − F)/I) × 100; retained as (I − (F + U)); and retained (%) R/I as (I − (F + U))/I × 100, where I is intake, F is fecal excretion, and U is urinary excretion.

The Ethics Committee on Animal Experimentation of the University of Granada (Reference: CEEA 2011-356) approved all the experiments, which were developed according to the Directional Guides Related to Animal Housing and Care (European Council Community, 1986).

### 2.2. Determination of Total Metals

For determining the metals, the samples were previously lyophilized. Subsequently, metals were quantified according to the protocol described by our research group [29,31]. Determination of V, Zn, Cu and Mn in the diet, serum and tissues was performed using inductively coupled plasma spectroscopy–MS (ICP-MS, He/H2 mode) (Agilent 7500; Agilent Technologies, Tokyo, Japan). The samples were then digested with nitric acid and hydrogen peroxide in a microwave oven (Milestone, Sorisole, Italy). After sample digestion, the extract was collected and made up to a final volume of 10 mL for subsequent analysis. Calibration curves were prepared following the Ga addition technique as an internal standard, using stock solutions of 1000 mg/L of each element (Merck) [29,31]. The accuracy of the method was evaluated by analyzing five independent determinations of suitable certified reference materials, Seronorm (Billingstad, Norway) and NIST 8414 (Gaithersburg, MD, USA), and by recovery studies on samples of organs enriched with multi-element standards. The coefficient of variation percentages obtained for V, Zn, Cu and Mn were 5.6, 2.4, 2.6 and 2.9, respectively.

### 2.3. Analysis of DMT1 Gene Expression in Liver Samples by RT-qPCR

The DMT1 gene expression in the liver was determined following the protocol described by Sánchez-González et al. (2017) [32] with the following primer sequences: Forward 5′-CATGCTTTACCGGTCAACTACATC-3′, Reverse 5′-TCACAGTTTGGAGCAGCACTTG-3′.

### 2.4. Determination of Antioxidant Enzyme Activity and Proinflammatory Parameters

Methodology for glutathione peroxidase (GPx), glutathione reductase (GR); reduced glutathione (GSH), glutathione transferase (GST), superoxide dismutase (SOD), catalase (CAT), NAD(P)H: quinone-oxidoreductase-1 (NQO1), protein carbonyl group and malondialdehyde (MDA) determination have previously been described [33,34]. Moreover, at day 35, proinflammatory parameters (leptin, C-reactive protein (CRP), interleukin-6 (IL-6), interleukin-1β (IL-1β) and tumor necrosis factor-alpha (TNF-α)) were determined in serum following the methodology proposed by Sánchez-González et al. [14].

### 2.5. Statistical Analysis

Mean and standard deviation were determined for each variable studied. Statistical significance was evaluated using one-way analysis of variance (ANOVA) followed by Bonferroni post hoc test for multiple comparisons. For bivariate analysis, Spearman’s correlation coefficient was calculated. All the analyses were performed using Statistical Package for Social Science 20.0 (SPSS, Chicago, IL, USA).

## 3. Results

The doses used for this experiment are supported by previous studies conducted by our research group [35,36]. In addition, two rats from each group of rats treated with BMOV(IV) were withdrawn from the study due to gastrointestinal disorders. The relative information regarding food intake, weight, water and glucose index has previously been summarized [33,35].

Table 1 shows the digestive and metabolic parameters of Zn, Cu and Mn during the last week of treatment. Treating hyperglycemic rats with two doses of V promoted the same trends for all the metallic elements studied. The food intake (and, therefore, Cu, Zn and Mn intake) in H and HV rats was higher than in the C group, although the increase in the HV group was lower than in the H group. Fecal excretion of the three elements was related to intake. However, urinary excretion was different for each element: Zn and Mn urinary excretion in the H and HV groups were increased compared to the C group. Copper urinary excretion in the H and HV group was similar to that of the C group.

The levels of the three absorbed and retained elements follow a behavior similar to the intake and fecal excretion, being higher than the control in both groups (H and HV). The absorbed and retained Zn, Cu and Mn percentages of H and HV show no significant changes compared to the C group.

Treatment with 3 mg V/day (HVH group) generally tends to recover the deviations found in the H and HV groups and approximate the control values (Table 1).

Table 2 summarizes the content of each mineral in the serum and tissues determined by ICP-MS. The H group showed an increase in Zn and Cu content in the liver, kidneys, serum and femurs, and no changes in Mn content compared to the C group. Treatment with 1 mg V/day (HV) showed no significant changes compared with the H group, except for hepatic Cu. Copper follows a similar pattern, its content being higher in the liver, serum and kidneys of the HV group than in the C group. Mn has no significant changes in the HV group compared to the C group, except for the heart, where it decreases. Exposure to 3 mg V/day (HVH group) tends to correct the effects caused by diabetes by approximating the concentrations of both elements to the control levels, including significant decreases in serum levels of Zn and Mn and kidney and liver levels of Zn. The 3 mg V/day supplementation also decreases Cu content in the kidney compared to H and HV groups, but it is higher than in the C group (Table 2).

**Figure 2:**
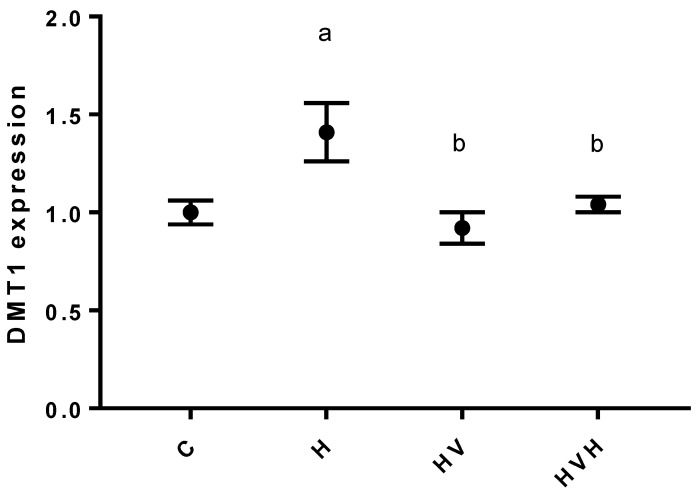
Changes in DMT1 expression in kidney tissue. *p* < 0.05. C (control rats); H (hyperglycemic rats); HV (hyperglycemic rats treated with 1 mg V per day); HVH (hyperglycemic rats treated with 3 mg V per day). (a) vs. control; (b) vs. H. *p* < 0.05.

Figure 2 shows the relative expression of the DMT1 transporter. Diabetes significantly increased the expression of this transporter, and supplementation with V (1 mg V/day and 3 mg V/day) promoted its decrease.

Table 3 summarizes a bivariate analysis using the Spearman correlation test. In this case, the relationship between food intake and serum and tissue levels of each metallic element was explored.

Table 4 shows Spearman’s rank correlation between serum and liver levels of Zn, Cu and Mn with oxidative status in the liver. Moreover, the activities of glutathione peroxidase (GPx), glutathione reductase (GR), glutathione transferase (GST), superoxide dismutase (SOD), catalase (CAT), NAD(P)H: quinone-oxidoreductase-1 (NQO1), protein carbonyl group levels and malondialdehyde (MDA) levels in the liver at day 35 were previously determined by our research group [33,34].

Table 5 shows Spearman’s rank correlation between serum levels of Zn, Cu and serum proinflammatory parameters. The levels of proinflammatory parameters, leptin, C-reactive protein (CRP), interleukin-6 (IL-6), interleukin-1β (IL-1β) and tumor necrosis factor-alpha (TNF-α) were determined in serum at day 35. These results are summarized in previous literature [14].

## 4. Discussion

Diabetes is a disease affecting trace element metabolism, which may contribute to the development of the disease. V is a trace element linked to glucose regulation, improving its transport and metabolism and increasing the sensitivity of the insulin receptor [37]. However, some aspects need to be clarified, such as the interactions with other trace elements. Hence, the present study examined hyperglycemic rats treated with different doses of V to determine Zn, Cu and Mn bioavailability, tissue distribution and their relationship with some indicators of the nutritional status of these elements.

The evolution of this disease, regarding weight changes, food and water intake, urinary excretion and serum insulin, was previously published by our research group [32,33]. In that case, the H group showed signs of pathology: hyperphagia, polydipsia, polyuria and hyperglycemia. Treatment with 1 mg V/day (HV group) produced a decrease in food intake but did not significantly modify water intake or urinary excretion and showed no hypoglycemic effect regarding the H group. On the other hand, rats treated with 3 mg V/day showed lower values for water intake, urinary excretion and glycaemia, similar to those of the control [32,33]. Therefore, disease progress is similar to treatment with insulin, thus, reducing hyperglycemia, hyperphagia and polydipsia.

At the conditions studied, hyperphagia in the H group, which is associated with diabetes, could have promoted the increase in food intake [33] and, consequently, increased Zn, Cu and Mn intake. Thus, this phenomenon could be associated with the increased net absorption of elements found in the H group.

Moreover, the sharp increase in urinary losses due to the polyuria caused by the altered endocrine state led to high urinary losses of Zn and Mn. However, loss of Cu showed no significant changes. The results obtained at the conditions studied were consistent with previous literature; the increase in urinary excretion of Zn but not Cu has previously been described [38]. Mn excretion in diabetic conditions is controversial because some authors showed that this disease increased its excretion [5,39], whereas others found no significant changes [38]. The high food intake could be associated with a significant increase in net mineral absorption and retention (Table 1). Hence, the digestive and metabolic changes found in the H group were caused by the polyphagia associated with this condition. The increase in Zn, Cu and Mn retention could be associated with the increase in Zn and Cu serum levels and the trend to increase Mn in hyperglycemic rats (Table 2).

Furthermore, when rats were treated with 1 mg V/day (group HV), this supplementation did not show any hypoglycemic effect, but reduced food intake, and consequently, the intake and urinary excretion of Zn, Cu and Mn was decreased compared to the H group [33]. No improvement was found in the metabolic parameters after treatment with V (Table 1). Thus, we can conclude that the HV group was similar to the H group; in fact, 1 mg V/day did not modify the serum levels compared to the H group (Table 2).

In contrast, treatment with 3 mg V/day (HVH) produced a clear hypoglycemic effect, normalizing glycemia and reducing the hyperphagia [33] and polydipsia present in the H group [34]; consequently, Zn, Cu and Mn intake were closer to the levels found in the C group. These effects decreased the mineral elements absorbed and retained, bringing them close to the control values and lower than those of the H and HV groups (Table 1).

The absence of significant changes in the % absorption and retention of elements in all groups suggested that the changes in the net absorption and retention of the cations were a consequence of food intake changes caused by the vanadium treatment (Table 1).

DMT1 is known to play an important role in the cellular uptake of Zn, Cu and Mn [3] and V (as V^3+^ or VO^2+^) [40]. However, the effect of V on the expression of this transporter is not fully understood. The present study evaluated the changes in the gene expression of DMT1 in liver tissue. The expression of DMT1 was elevated in the H group, and treatment with V (regardless of the dose used) decreased this expression to values similar to those in untreated rats (Figure 2). Ścibior et al. found no significant changes in DMT1 levels in the liver and the cerebral hemispheres of rats exposed to V(V) in the form of NaVO_3_ supplied in their drinking water, whereas the kidney levels of DMT1 decreased in these rats compared to control rats [27]. In contrast, other authors [41] showed increased DMT1 expression in human bronchial epithelial cells after exposition to V(IV) in the form of V(SO4)_2_. This transporter is expressed in enterocytes [3]. As mentioned above, in the present conditions, the intake changes in the three elements studied were motivated by their increased food intake (Table 1). This suggests that V exposition did not alter the expression of DMT1 in enterocytes. In previous studies, no changes were found in the intestinal absorption of Mn in hyperglycemic rats treated with 1 mg V/day (BMOV(IV)) [29].

Serum levels of Zn and Cu in the H and HV groups were higher than those in the C group because the animals in these groups consumed more food, which increased the net values of Zn, Cu and Mn absorbed and retained (Table 1). The increased body retention of Zn and Cu caused by diabetes is assumed to be responsible for the increased serum levels, and the content of these cations in the tissues studied (Table 2). The correlations found between food intake (and, therefore, element intake) and tissue content (Table 3) corroborate this hypothesis. Thus, the higher food intake and increased DMT1 expression found in H tissue could be associated with the increase in mineral serum and tissue values. These results are consistent with other previously published data [42]. Regarding Mn, the H and HV groups increased net retention. Thus, Mn increased in serum and the liver, suggesting a positive correlation between intake and the Mn content in these tissues (Table 3). Karganov et al. described how Mn was deposited in the liver (without changes in other tissues, such as serum or spleen) in diabetic rats [42]. Furthermore, V supplementation enhanced Mn uptake in HepG2 cells [20].

In previous research, an association was found between the increase in trace elements and the increase in food intake, but tissue distribution is not the same for all the minerals. For example, the liver and kidneys are normally the tissues where these increases are higher [43,44,45,46]. These findings are consistent with the results obtained in the present study; in this case, when food intake was increased, Zn tended to be the mineral where tissue deposits increased more, followed by Cu, whereas no significant changes were found in Mn (Table 2).

No hypoglycemic effects were observed when supplementing rats with 1 mg V/day (HV), despite the decrease in food intake [33]. Even though no significant differences in the mineral content of the tissues studied were found between H and HV groups, there was a trend towards decreased content after supplementation, especially for Zn in the liver, kidney, spleen and femur. This could be facilitated by the decrease in DMT1 expression in this group. In contrast, exposing the rats to 3 mg V/day (HVH) had a clear hypoglycemic effect, normalizing the glycemic levels and reducing food intake to levels similar to those of the control [33]. Moreover, this V dose severely reduced the net absorption of three elements due to reduced intake (Table 1). In the HVH group, we believe that decreased absorption is responsible for the lower Zn, Cu and Mn content in serum and Zn and Cu in the kidneys and Zn, Cu and Mn in the liver and spleen (Table 3). Moreover, the decrease in DMT1 expression may facilitate this mineral decrease. Other authors described the effect of V on hyperglycemic rats, but differences could be attributed to doses or chemical compounds.

In identical working conditions, our research group described how treating rats with BMOV(IV) altered the homeostasis of Se [33], Mg [36] and Fe [34] and that these alterations were associated with changes in the antioxidant status. We also discovered that V treatment causes changes in serum levels of proinflammatory cytokines [14]. In the liver, we found that BMOV(IV) increased the activity of catalase (CAT), protein carbonyl groups and malonaldehyde (MDA) levels. Moreover, the inclusion of V decreased the activity of GPx, GST and NQO1 and did not alter the SOD and GR [33,34,36]. The present study aimed to find out whether the changes found in these enzyme activities are related to the changes in the hepatic content of Zn, Cu and Mn caused by treating hyperglycemic rats with BMOV(IV). There is a wealth of literature on the relationship between antioxidant status and Zn, Cu and Mn content in various tissues. However, inconsistent results were found in the literature. Thus, supplementation with Fe, Cu, Mn and Zn increased the deposits of Zn in muscle, and these increases are positively related to GPx and SOD and a decrease in oxidative stress [47]. Zhou et al. described how, after exposing mussels to different metals, the content of Zn in the mantle was negatively associated with the activity of GPx, finding no relation with SOD and CAT, while the contents of Cu and Mn were not correlated with any of the three enzymes mentioned [11]. In glioma C6 cells, exposure to Cu (II) complexes increased SOD and CAT activity, but not GPx activity [48]. After in-vitro liver and kidney exposure to Cd, Cu and Zn, increased GPx activity was found, whereas CAT and SOD levels decreased, with Zn being more effective in the liver and Cu in the kidneys [49]. Studies conducted on fish reported that supplementation with Cu nanoparticles increased GPx, SOD and CAT activities and decreased MDA levels [50]. Gao et al. found a positive correlation between plasma Zn content and GPx activity and a negative correlation between plasma Zn content and MDA content [51]. However, other authors negatively related serum Zn to GPx and positively to SOD and found no significant relationship with CAT and MDA content, whereas serum Cu content positively relates to GPx and negatively relates to SOD and CAT; no significant relationship with MDA was found [52]. It has been described how Zn supplementation increases SOD activity but has no effect on GPx activity [53]. In chickens supplemented with zinc sulfate or zinc oxide nanoparticles, no liver increases in the gene expression of SOD, CAT, GST, GPx and NQO1 have been found [45]. However, it has been reported that exposing fish to Zn oxide nanoparticles increased SOD, CAT, GST and GPx activity [46]. Other authors found no changes in SOD and GPx after supplementation with Zn, Cu, Se and Mn supplied by subcutaneous injection to dairy cows [54]. The results of our study show that the hepatic content of Zn, Cu and Mn is positively correlated with GPx activity and negatively correlated with CAT activity. In contrast, Zn and Mn appear to be directly correlated with NQO1 and negatively correlated with MDA levels (Table 5). These associations suggest that the changes in tissue contents caused by the treatment could alter the oxidative state described in the studies mentioned above. However, in our case, SOD, GR and GST activity was not associated with the liver content of these metals (Table 5).

Regarding proinflammatory cytokines, we found that the V treatment modified the proinflammatory levels, decreasing leptin and increasing CRP and interleukin-6 (IL6) and did not modify interleukin 1β (IL-1β) and tumor necrosis factor-alpha (TNF-α) [14]. The present study did not significantly correlate changes in serum levels of Zn, Cu and Mn with the proinflammatory parameters, except for the negative association between leptin and Cu levels (Table 5). Previous studies reported inconsistent effects; some authors described how exposing bronchial epithelium cells (BEAS-2B) to metals promoted different changes in the proinflammatory homeostasis, i.e., exposition to Zn and Mn induced IL-6 liberation, but Cu supplementation did not modify it [55]. Li et al. described how this Cu exposure significantly increased the TNF-α in chickens [56]. In contrast, exposing turkeys to Mn (as Mn oxide or nanoparticles) decreased IL-6 levels [43]. Decreases in IL-β and TNF-α gene expression in the liver of “Nile tilapia”, fed diets supplemented with Zn or Cu, have been observed [44]. Recently, in patients with rheumatoid arthritis, serum CRP levels have been positively associated with Cu levels but not with Zn and Mn levels [57]. The information above is consistent, in part, with the results found in our study, as a result of treating hyperglycemic rats with BMOV(IV). The differences observed could be due to the different conditions of the experiments, species used and tissues used for the determinations.

## 5. Conclusions

The results acquired from the present study showed that BMOV(IV) administered as a hypoglycemic agent at a dose of 3 mg V per day to hyperglycemic rats has a very beneficial effect on Zn and Cu homeostasis and that its effect is mediated by reduced food intake and DMT1 expression. This reduction reverted the alterations in Zn and Cu homeostasis produced by diabetes mellitus. Nevertheless, more studies are required to better determine the mode of action responsible for the effects observed, to study the effects of these interactions, to evaluate the optimum level of pharmacological intervention and to avoid or prevent side effects.

## Figures and Tables

**Table 1:** Digestive and metabolic utilization of Zn, Cu and Mn on days 28–35 of study.

Group	C (*n* = 9)	H (*n* = 8)	HV (*n* = 8)	HVH (*n* = 8)	P_ANOVA_
**Zn**					
Intake (I) (µg/day)	609 ± 80	1342 ± 95 ^a^	1090 ± 81 ^a,b^	560 ± 45 ^b,c^	*p* < 0.001
Fecal excretion (F) (µg/day)	294± 49	606 ± 177 ^a^	473 ± 107 ^a^	259 ± 62 ^b,c^	*p* < 0.001
Urinary excretion (U) (µg/day)	3.8 ± 1.3	40 ± 5 ^a^	34 ± 2 ^a,b^	5.6 ± 2.9 ^b,c^	*p* < 0.001
Absorbed (A); A = I − F (µg/day)	323 ± 85	735 ± 145 ^a^	615 ± 126 ^a^	302 ± 57 ^b,c^	*p* < 0.001
%Absorbed; %A = (A × 100/I)	52 ± 9	55 ± 12	56 ± 10	54 ± 10	NS
Retained (R); R = [I − (F/U)] (µg/day)	318 ± 84	681 ± 136 ^a^	586 ± 127 ^a^	297 ± 59 ^b,c^	*p* < 0.001
%Retained; %R = (R × 100/I)	51 ± 9	51 ± 11	54 ± 10	53 ± 10	NS
**Cu**					
Intake (I) (µg/day)	93 ± 12	206 ± 14 ^a^	168 ± 12 ^a,b^	86 ± 7 ^b,c^	*p* < 0.001
Fecal excretion (F) (µg/day)	55± 10	109 ± 10 ^a^	83 ± 20 ^a,b^	44 ± 9 ^b,c^	*p* < 0.001
Urinary excretion (U) (µg/day)	3.4 ± 0.9	4.1 ± 1.3	2.l ±1. 2	8 ± 6 ^a,c^	*p* < 0.01
Absorbed (A); A = I − F (µg/day)	39 ± 9	98 ± 19 ^a^	85 ± 21 ^a^	41 ± 8 ^b,c^	*p* < 0.001
%Absorbed; %A = (A × 100/I)	41 ± 9	47 ± 7	50 ± 11	48 ± 9	NS
Retained (R); R = [I − (F/U)] (µg/day)	35 ± 9	93 ± 19 ^a^	82 ± 21 ^a^	33 ± 9^,b,c^	*p* < 0.001
%Retained; %R = (R × 100/I)	38 ± 9	45 ± 7	49 ± 10^a^	38 ± 13	NS
**Mn**					
Intake (I) (µg/day)	192 ± 25	415 ± 183 ^a^	290 ± 101 ^a,b^	179 ± 33 ^b,c^	*p* < 0.001
Fecal excretion (F) (µg/day)	124 ± 25	248 ± 73 ^a^	188 ± 47 ^a^	95 ± 17 ^b,c^	*p* < 0.001
Urinary excretion (U) (µg/day)	0.08 ± 0.2	0.33 ± 0.20 ^a^	0.37 ± 0.22 ^a^	0.13 ± 0.11 ^b,c^	*p* < 0.001
Absorbed (A); A = I − F (µg/day)	68 ± 17	181 ± 61 ^a^	152 ± 41 ^a^	84 ± 19 ^b,c^	*p* < 0.001
% Absorbed; %A = (A × 100/I)	35 ± 9	42 ± 16	44 ± 12	46 ± 9	NS
Retained (R); R = [I − (F/U)] (µg/day)	67 ± 17	180 ± 61 ^a^	151 ± 41 ^a^	83 ± 19 ^b,c^	*p* < 0.001
%Retained; %R = (R × 100/I)	35 ± 9	42 ± 16	44 ± 12	46 ± 9	NS

The values shown are means ± SD. C (control rats); H (hyperglycemic rats); HV (hyperglycemic rats treated with 1 mg V per day); HVH (hyperglycemic rats treated with 3 mg V per day). (^a^) vs. control rats (C); (^b^) vs. hyperglycemic rats (H); (^c^) vs. hyperglycemic rats treated with 1 mg V per day (HV). *p* < 0.05. NS = not significant).

**Table 2:** Vanadium, zinc, copper and manganese content in serum (µg/L) or dry tissue (mg/kg) from the kidney, liver, skeletal muscle, spleen, heart and femur on day 35.

	Control (*n* = 9)	H (*n* = 8)	HV (*n* = 8)	HVH (*n* = 8)	P_ANOVA_
***Serum***					
V	2.4 ± 0.5	6.2 ± 1.3	385 ± 96 ^a,b^	766 ± 202 ^a,b,c^	*p* < 0.001
Zn	1505 ± 139	1729 ± 82 ^a^	1783 ± 191 ^a^	1331 ± 162 ^b,c^	*p* < 0.001
Cu	851 ± 117	1020 ± 190 ^a^	1027 ± 132 ^a^	948 ± 95	*p* < 0.05
Mn	1.6 ± 0.4	1.9 ± 0.3	1.9 ± 0.9	1.0 ± 0.3 ^b,c^	*p* < 0.05
***Kidney***					
Zn	101 ± 12	140 ± 17 ^a^	128 ± 19 ^a^	79 ± 10 ^a,b,c^	*p* < 0.001
Cu	23 ± 6,3	159 ± 28 ^a^	162 ± 20 ^a^	69 ± 15 ^a,b,c^	*p* < 0.001
Mn	3.5 ± 0.48	3.6 ± 0.36	3.6 ± 0.37	3.3 ± 0.30	NS
***Liver***					
Zn	121 ± 18	148 ± 19 ^a^	141 ± 18	69 ± 7 ^a,b,c^	*p* < 0.001
Cu	15 ± 1.4	24 ± 5,6 ^a^	17 ± 2.1 ^b^	15 ± 1.7 ^b^	*p* < 0.001
Mn	8.2 ± 1.2	8.4 ± 1.2	9.8 ± 2.2	6.1 ± 0.83 ^b,c^	*p* < 0.01
***Skeletal muscle***					
Zn	36 ± 4.8	46 ± 8.4	57 ± 24 ^a^	39 ± 4	*p* < 0.01
Cu	2.8 ± 0.45	2.9 ± 0.45	2.7 ± 0.19	2.7 ± 0.33	NS
Mn	0.29 ± 0.08	0.32 ± 0.10	0.26 ± 0.09	0.26 ± 0.05	NS
***Spleen***					
Zn	76 ± 3.8	80 ± 3.4	77 ± 5	75 ± 10	NS
Cu	1.9 ± 0.11	2.2 ± 0.11	2.2 ± 0.30	2.0 ± 0.22	*p* < 0.05
Mn	0.63 ± 0.05	0.65 ± 0.04	0.61 ± 0.09	0.56 ± 0.06	NS
***Heart***					
Zn	67 ± 8.4	65 ± 9.0	63 ± 3	61 ± 5	NS
Cu	12 ± 1.5	12 ± 2.0	12 ± 0.82	11 ± 1.4	NS
Mn	1.2 ± 0.14	1.1 ± 0.22	0.97 ± 0.05 ^a^	1.1 ± 0.14	*p* < 0.05
***Femur***					
Zn	66 ± 2.7	82 ± 8.7 ^a^	72 ± 16	69 ± 10 ^b^	*p* < 0.05
Cu	0.42 ± 0.04	0.52 ± 008 ^a^	0.45 ± 0.10	0.44 ± 0.04	*p* < 0.05
Mn	0.26 ± 0.02	0.28 ± 0.04	0.24 ± 0.07	0.25 ± 0.04	NS

The values shown are means ± SD. C (control rats); H (hyperglycemic rats); HV (hyperglycemic rats treated with 1 mg V per day); HVH (hyperglycemic rats treated with 3 mg V per day). (^a^) vs. control group (C); (^b^) vs. hyperglycemic rats (H); (^c^) vs. hyperglycemic rats treated with 1 mg V per day (HV). *p* < 0.05. NS = not significant).

**Table 3:** Spearman’s rank correlation coefficient between serum levels of V, Zn, Cu and Mn and levels of Zn, Cu and Mn in tissue.

Tissue		Food Intake
Serum	V	−0.390 ^a^
	Zn	0.712 ^a^
	Cu	0.423 ^a^
	Mn	0.482 ^a^
Kidney	Zn	0.788 ^a^
	Cu	0.876 ^a^
	Mn	NS
Liver	Zn	0.703 ^a^
	Cu	0.664 ^a^
	Mn	0.430 ^a^
Spleen	Zn	NS
	Cu	0.499 ^a^
	Mn	NS
Skeletal muscle	Zn	0.375 ^a^
	Cu	NS
	Mn	NS
Heart	Zn	NS
	Cu	NS
	Mn	NS
Femur	Zn	0.510 ^a^
	Cu	0.415 ^a^
	Mn	NS

(^a^) *p* < 0.05. NS = not significant.

**Table 4:** Spearman’s rank correlation coefficient between liver antioxidant enzymes activity and Zn, Cu and Mn liver levels.

Liver Antioxidant Enzymes Activity	Liver Zn	Liver Cu	Liver Mn
GPx	0.798 ^a^	0.658 ^a^	0.529 ^a^
GX	NS	NS	NS
GST	NS	NS	NS
SOD	NS	NS	NS
CAT	−0.796 ^a^	−0.446 ^a^	−0.497 ^a^
NQO1	0.536 ^a^	NS	0.475 ^a^
Protein carbonyl groups	NS	NS	NS
MDA	−0.666 ^a^	NS	−0.451 ^a^

(^a^) *p* < 0.05. NS = not significant. Glutathione peroxidase (GPx), glutathione reductase (GR); reduced glutathione (GSH), glutathione transferase (GST), superoxide dismutase (SOD), catalase (CAT), NAD(P)H: quinone-oxidoreductase-1 (NQO1), protein carbonyl group and malondialdehyde (MDA).

**Table 5:** Spearman’s rank correlation coefficient between serum levels of proinflammatory parameters and Zn, Cu and Mn.

Serum Proinflammatory Cytokines	Serum Zn	Serum Cu	Serum Mn
Leptin	NS	−0.468 ^a^	NS
CRP	NS	NS	NS
IL β	NS	NS	NS
IL-6	NS	NS	NS
TNF α	NS	NS	NS

(^a^) *p* < 0.05. NS = not significant. C-reactive protein (CRP), interleukin-6 (IL-6), interleukin-1β (IL-1β), and tumor necrosis factor-alpha (TNF-α).

## Data Availability

Not applicable.
